# Call on me! Undergraduates’ perceptions of voluntarily asking and answering questions in front of large-enrollment science classes

**DOI:** 10.1371/journal.pone.0243731

**Published:** 2021-01-12

**Authors:** Erika M. Nadile, Emilie Alfonso, Briana Michelle Barreiros, William D. Bevan-Thomas, Sara E. Brownell, Megan R. Chin, Isabella Ferreira, Sariah A. Ford, Logan E. Gin, Jomaries O. Gomez-Rosado, George Gooding, Alyssa Heiden, Airyn E. Hutt, Meagan L. King, Shannon G. Perez, Yasiel I. Rivera Camacho, Flor Salcedo, Christopher F. Sellas, Krystian A. Sinda, Katherine N. Stahlhut, Michelle D. Stephens, Nicholas J. Wiesenthal, Keonti D. Williams, Yi Zheng, Katelyn M. Cooper

**Affiliations:** 1 The Biology Education Research Lab, Research for Inclusive STEM Education Center, School of Life Sciences, Arizona State University, Tempe, AZ, United States of America; 2 BSC 4932: Undergraduate Biology Education Research Class, Department of Biology, University of Central Florida, Orlando, FL, United States of America; 3 Mary Lou Fulton Teachers College, Arizona State University, Tempe, Arizona, United States of America; 4 The Department of Biology, University of Central Florida, Orlando, FL, United States of America; University of Eastern Finland, SWEDEN

## Abstract

Allowing students to voluntarily ask and answer questions in front of the whole class are common teaching practices used in college science courses. However, few studies have examined college science students’ perceptions of these practices, the extent to which students choose to engage in these practices, and what discourages students from participating. In this study, we surveyed 417 undergraduates at a research-intensive institution about their experiences asking and answering questions in large-enrollment college science courses. Specifically, students answered questions about to what extent they perceive voluntarily asking and answering questions in large-enrollment science courses is helpful to them and why. They also answered questions about to what extent they engage in asking and answering questions in large-enrollment college science courses and what factors could discourage them from participating. Using binary logistic regression, we examined whether there were differences among students of different demographic groups regarding their opinions about asking and answering questions. We found that overwhelmingly students reported that other students voluntarily asking and answering instructor questions is helpful to them. Notably, compared to continuing generation students, first-generation students were more likely to perceive other students asking questions to be helpful. Despite perceiving asking and answering questions to be helpful, over half of students reported that they never ask or answer questions in large-enrollment college science courses during a semester, and women were more likely than men to report never asking questions. We identified fear of negative evaluation, or students’ sense of dread associated with being unfavorably evaluated, as a primary factor influencing their decision to answer instructor questions. This work adds to a growing body of literature on student participation in large-enrollment college science courses and begins to uncover underlying factors influencing student participation.

## Introduction

Eliciting student participation by giving students the opportunity to ask or answer questions in front of the whole class is a common way for instructors to engage students in the college science classroom [1–8]. Sometimes dubbed the “Socratic method” of teaching, studies have shown that encouraging students to ask and answer questions is frequently employed in both small-enrollment and large-enrollment college courses [5,9–14]. Despite the near ubiquitous nature of giving students the opportunity to voluntarily ask and answer questions in front of the class, there are few studies that have examined the benefits of this type of participation specifically in the context of college science courses. More commonly, studies have examined the impact of “student participation,” a broad term describing students’ contributions to whole-class discussion; this encompasses answering and asking questions, as well as contributing dialogue to whole-class conversations or debates.

Student participation in whole-class discussions across college disciplines has been linked to an array of positive outcomes for the student participating, including enhanced learning and critical thinking [15–20]. In the context of college science courses, students who voluntarily participated in a large-enrollment discussion-style chemistry course showed greater learning gains on a chemistry concept inventory compared to those who did not frequently participate [7]. Additionally, college students across disciplines report that giving students the opportunity to participate during class can create the feeling of a comforting, warm classroom environment, one in which students feel as though the instructor cares and that they have a perceived relationship with the instructor [14,21–25].

While the literature on having students engage in dialogue in front of the whole class focuses primarily on how this practice can benefit students, there is some evidence to suggest that this practice could, at times, be detrimental to students in college science courses. Questions that are off-topic or too advanced may actually confuse some students in the course [26], leading to tangential whole-class conversations that are less relevant to the learning goals of the course and potentially wasting valuable class time. Additionally, students can also become aggravated when a subset of students ask too many questions or dominate class discussions [5]. Finally, studies have suggested that some students may experience anxiety if they are required to participate in whole-class discussions [27–30] and that even volunteering to answer questions can exacerbate some students’ anxiety [29]. As such, more research is required to further understand the extent to which participating in class could potentially negatively affect students.

Inequitable participation, or the differences in which students engage in whole-class discussion, is also a potential problem in college science classrooms that may negatively affect groups of students. Previous studies have suggested that certain social identity groups, including women, Black students, and students with anxiety, are less likely to participate in class [10–12,31–40]. For instance, studies have shown that women are less likely to participate in whole-class discussions in large-enrollment college science courses compared to men [10–12,31–34]. While researchers have suggested that women’s lack of confidence and the “chilly” environment of college science classrooms may partially explain this gap, few studies have documented what factors explain these gender differences [35–39]. Differences in participation among students of different races/ethnicities is less established. A study of primarily first-year students in a large-enrollment introductory biology course found that Black students were significantly less likely to self-report that they speak up in a traditional lecture class compared to white students [40]. However, Black students reported speaking up at the same rate as white students in active learning classes where they engaged in activities to enhance their learning. The authors hypothesized that Black students’ increased participation may have been because of an increased sense of community in the active learning classes, but they did not ask the students whether this contributed to their participation. Finally, studies have demonstrated that students with high anxiety, particularly those who fear being negatively evaluated or judged by others, report feeling highly uncomfortable participating in whole-class discussions, which they report can decrease their willingness to engage in such activities [27–30]. The potential for demographic differences in participation suggests that students may have inequitable experiences when engaging in college science courses. Specifically, students may have differing perceptions of the competence of their peers based on demographics [41–43] and may experience differences in learning gains [7]. However, researchers have only examined participation differences among a few demographic groups, and limited studies have examined whether there are participation gaps specifically with asking and answering questions during whole-class discussions.

While studies across disciplines suggest that voluntary student participation in college science courses can benefit students, we know of no studies that have explored students’ perceptions of two specific participation practices: asking and answering questions in front of the whole class. In this study, we explored students’ perceptions of voluntarily asking and answering questions in front of the whole class in large-enrollment college science courses and whether student demographics predicted their opinions and experiences.

Our specific research questions are:

1. To what extent do undergraduates perceive that others voluntarily **asking questions** in large-enrollment science courses is helpful and why?
2. How frequently do undergraduates report personally **asking questions** in large-enrollment science courses during a semester and what discourages students from asking questions?
3. To what extent do undergraduates perceive that others voluntarily **answering questions** in large-enrollment science courses is helpful and why?
4. How frequently do undergraduates report personally **answering questions** in large-enrollment science courses during a semester and what discourages students from answering questions?

For each of these questions we examined whether student demographics predicted their self-reported experiences.

## Methods

This study was done with approved Institutional Review Board protocols: #1519 from the University of Central Florida and #11614 from Arizona State University.

This study was conducted as part of a semester long course-based undergraduate research experience (CURE) taught by K.M.C. at the University of Central Florida in spring 2020. In a CURE, students engage in a research project that is novel and broadly relevant in the context of a course [44–46]. The CURE was offered by the Department of Biology and was backward designed to improve students’ process of science and quantitative reasoning skills [46,47]. Specifically, students engaged in a novel, broadly relevant research project with the intent to improve their scientific thinking, information literacy, question formulation, study design, data analysis, interpretation, and evaluation skills [48]. Nineteen undergraduates were enrolled in the CURE and were researchers on this project. Collectively with the instructor, the students were responsible for developing the research questions, creating the measurement tool, analyzing the data, interpreting the data, and communicating the findings. The first half of this course was taught in-person and second half of the course was taught online due to the COVID-19 pandemic; all data for the project were collected prior to universities delivering courses online due to COVID-19.

### Survey development

In fall 2019, E.M.N. conducted 50 exploratory semi-structured interviews with undergraduates to begin to understand what affects students’ willingness to voluntarily ask and answer questions in large-enrollment college science courses. The 50 interview participants were recruited from two upper-level large-enrollment biology courses at a large institution with highest-research activity (R1) in the Southwest United States. The demographics of the interview participants and a copy of the interview script are reported in the S1 Appendix. The data from these interviews served as pilot data for the current study and informed the survey development in spring 2020.

The survey created in spring 2020 was developed to further understand (a) to what extent undergraduates perceive voluntarily asking and answering questions in large-enrollment science courses to be helpful and why, and (b) to what extent undergraduates engage in asking and answering questions in large-enrollment science courses and why. To establish cognitive validity of the survey, twenty-one researchers reviewed and modified the survey questions using criteria developed to assess each question (e.g. Is the meaning of this question clear? Is this question grammatically correct?) [49]. Then, 19 researchers conducted individual think-aloud interviews with a total of 19 undergraduate science students to ensure that students understood what each question was asking [50]. The researchers compiled their notes from the interviews and revised the survey. Nineteen researchers conducted a second set of think-aloud interviews using the revised survey with an additional 19 undergraduate science students, compiled their interview notes, and revised the survey. Twenty-one researchers then reviewed the revised survey and provided a final set of recommended revisions before the survey was revised a final time. The survey was revised a total of four times based on 80 instances of individual feedback. A copy of the survey questions that were analyzed is provided in the S1 Appendix.

#### Screening

At the beginning of the survey, we defined large-enrollment college science courses as courses in biology, chemistry, geosciences, or physics with 100 students or more. Given this definition, we asked survey respondents whether they had ever been enrolled in a large-enrollment college science course where students were able to voluntarily ask questions and also whether they had ever been enrolled in a large-enrollment college science course where students were able to voluntarily answer questions. Any student who had not been enrolled in both a large-enrollment course where they were able to voluntarily ask questions and a large-enrollment course where they were able to voluntarily answer questions was excluded from the study.

#### Examining to what extent students perceive other students voluntarily asking questions is helpful in large-enrollment college science courses

We asked students to what extent they feel it is helpful, on average, when they hear other students voluntarily ask the instructors questions in large-enrollment college science courses, which they answered on a Likert-scale ranging from 1 (extremely unhelpful) to 6 (extremely helpful). After answering this closed-ended question, all students were asked an open-ended question prompting them to provide reasons why they felt hearing other students voluntarily ask questions was helpful. Using data collected from the pilot interview study, we had previously identified five reasons why students felt hearing others ask questions was helpful. Next, we presented students with these five reasons and asked them to select any of the reasons they felt hearing other students voluntarily ask questions in large-enrollment college science courses was helpful to them. Students also had the option of selecting “none of these reasons apply to me.”

#### Examining students’ perceptions of how frequently they ask questions during a semester and what discourages them from asking questions

Students were asked, on average, how often they voluntarily ask questions to instructors in front of large-enrollment college science courses during one semester. Students answered the question with four choices: Never (0 questions per semester), not often (1–2 questions per semester), somewhat often (3–4 questions per semester), and fairly often (5 or more questions per semester). Students were then presented with an open-ended question asking them to report what factors discourage them from voluntarily asking questions to instructors in front of the whole class in large-enrollment college science courses. Using the pilot interview data, we had previously identified nine reasons why students reported they were discouraged from voluntarily asking questions in large-enrollment college science courses. After students answered the open-ended question, we presented students with the nine pre-determined reasons and asked them to select all that would discourage them from voluntarily asking questions. Students also had the option of selecting “none of these reasons apply to me.”

#### Repetition of survey questions for answering questions

We developed a similar set of questions to probe students’ thoughts about voluntarily answering instructor-posed questions in front of the whole class in large-enrollment college science courses. Students were asked about answering questions after they completed the set of items about asking questions.

#### Demographics

At the end of the survey, students answered a suite of demographic questions asking about their gender, race/ethnicity, college generation status, year in college, and grade-point-average (GPA). Additionally, we hypothesized that students’ comfort speaking English may influence the extent to which they benefited from hearing questions being asked and answered and to what extent they themselves voluntarily asked and answered questions in front of the whole class [2,51,52]. As such, we asked students a Likert-scale question about how comfortable they felt speaking English in front of others, ranging from 1 (extremely uncomfortable) to 6 (extremely comfortable). We also predicted that students’ fear of negative evaluation, or their sense of dread associated with being unfavorably evaluated while participating in a social situation [53,54], would affect students’ perceptions and behaviors regarding asking and answering questions [27,28,55]. We approximated students’ fear of negative evaluation by asking students to what extent they worry that others in their college science courses judge them when they interact, which they answered with 1 (never), 2 (rarely), 3 (sometimes), 4 (most of the time), and 5 (all the time).

### Survey distribution

An email was sent to instructors teaching biology, chemistry, physics, or geoscience courses at a large highest-research activity (R1) institution in the Southwest United States where the initial pilot study had been conducted. Of the 48 instructors who were contacted, 11 agreed to send the recruitment message and survey out to the students in their class. In exchange for taking the survey, students were offered a small amount of extra credit in the course from which they were recruited. In courses where students were unable to receive extra credit, they were entered into a drawing to win one of two $100 gift cards. All students were given approximately one week to complete the survey. Once all data were collected, students who did not consent to participate in the study, students who had never taken an in-person, large-enrollment college science course where they had the opportunity to voluntarily answer questions, and students who had never taken a large-enrollment college science course where they had the opportunity to voluntarily ask the instructor questions were removed from the dataset. In total, 417 students were included in the analyses and the data were deidentified. The number of students recruited from each science course are reported in the S1 Appendix.

### Analyses

#### Examining to what extent students perceive other students voluntarily asking and answering questions in large-enrollment college science courses is helpful and why

To assess whether there were demographic differences among the students who perceived other students asking questions to be helpful in large-enrollment college science courses, we pooled students’ responses to the question asking to what extent they perceive asking questions to be helpful; students who answered “extremely helpful,” “moderately helpful,” and “somewhat helpful” were grouped into the category “helpful” and students who answered “extremely unhelpful,” “moderately unhelpful,” and “somewhat unhelpful” were grouped into the category “unhelpful.” We then used binomial logistic regression to test whether there were demographic differences between students who considered others voluntarily asking questions to be helpful and students who considered others voluntarily asking questions to be unhelpful. We chose to include the following demographics in our model: gender (man, woman), race/ethnicity (Asian, white, Latinx), college generation status (first-generation, continuing generation), year in college (first year, second year, third year, fourth year or more), fear of negative evaluation (FNE), and GPA (Model: helpful/unhelpful ~ gender + race/ethnicity + college.generation + year.in.college + FNE + GPA). We recognize that not all students identify as gender binary (man or woman); however, there were too few students who identified as a gender other than man or woman to create a third category [56]. Owing to low sample sizes, we did not include students who identified as Black or African American, Pacific Islander, and American Indian or Alaska Native in the analyses. We intended to include student comfort speaking English in our model, however there was not enough variability in student responses to warrant the inclusion of this variable in the model. We chose not to include students’ majors in the model since nearly all students were science (e.g. biology) or science-related majors that require multiple science courses (e.g. nursing, kinesiology, engineering, psychology/neuroscience). There was no reason to think that different science majors would predict different outcomes since we asked students about their experiences across all science courses they had taken. We chose not to nest students within the classes they were recruited from because we did not exclusively recruit students from large-enrollment courses and we asked students to consider their average experiences in all of their large-enrollment college science courses.

To determine why students perceived asking questions to be helpful, we analyzed the data from the students who perceived asking questions to be helpful. A group of five researchers coded students’ responses to the open-ended question about why students perceived asking questions to be helpful using inductive coding [57]. The researchers concluded that the students’ open-ended responses reflected the closed-ended reasons why students might perceive others asking questions to be helpful that were developed from the pilot data. Because the open-ended responses generally reflected the closed-ended responses, we only report the analyses of the closed-ended responses in the manuscript for brevity (a more detailed description of how the open-ended data were coded, the categories that emerged from the open-ended responses, the percent of students who reported each category, and example quotes are reported in the S1 Appendix). We tallied the reasons that students checked as to why hearing other students asking questions in large-enrollment college science courses might be helpful to them. We used binomial logistic regression to test whether there were demographic differences in which students reported each reason (Model: reported reason yes/no ~ gender + race/ethnicity + college.generation + year.in.college + FNE + GPA). We ran an identical set of analyses to assess students’ responses to survey items probing their perceptions of answering questions.

#### Examining how frequently student report that they ask and answer questions during a semester and what discourages them from asking and answering questions

To assess whether there were demographic differences in the extent to which students reported voluntarily asking questions in large-enrollment college science courses, we pooled students’ responses into two categories: “never asked questions,” and “asked questions.” Students who answered that they answered “5 or more questions per semester,” “3–4 questions per semester,” or “1–2 questions per semester” were categorized as “asked questions” and students who reported never asking questions were categorized as “never asked questions.” We used binomial logistic regression to test whether there were demographic differences between students who reported voluntarily asking questions in front of college science courses and those who did not (Model: asked questions/never asked questions ~ gender + race/ethnicity + college.generation + year.in.college + FNE + GPA).

To determine what factors discouraged students from asking questions, we analyzed the data from the students who reported on average never asking questions or not often asking questions (asking 1–2 questions) in large-enrollment college science courses during a semester. A group of five researchers coded students’ responses to the open-ended question using inductive coding [57] to see whether students’ open-ended responses reflected the closed-ended reasons why they may be discouraged from asking questions developed from the pilot data. Because the open-ended responses generally reflected the closed-ended responses, we only report the analyses of the closed-ended responses in the manuscript (the categories that emerged from the open-ended responses, the percent of students who reported each category, and example quotes are reported in the S1 Appendix). We tallied the reasons that students checked as to why they themselves might be discouraged from asking questions in large-enrollment college science courses. We used binomial logistic regression to test whether there were demographic differences in which students reported the reason (Model: reported reason yes/no ~ gender + race/ethnicity + college.generation + year.in.college + FNE + GPA). We ran an identical set of analyses to assess students’ responses to survey items probing their perceptions about answering questions.

We recognize that the significance of a result from any statistical test is continuous rather than dichotomous based on the specific p-value [58]. However, we report select results by the criterion of p ≤ 0.05 throughout the results section for simplicity. We acknowledge that test results with p-values greater than 0.05 can still be scientifically meaningful, thus we report out all results of statistical tests in the S1 Appendix for the reader’s further interpretation. Additionally, we describe our results using language such as “women were 1.2x more likely than men to select a particular factor.” The number, in this case 1.2, is the natural exponential of the estimated coefficient for the explanatory variable, in this case “women” vs “men,” in the logistic regression model to predict whether the student will select a particular factor. This number is also called the “odds ratio,” which is a standardized effect size statistic in logistic regression [59,60].

## Results

Student demographics are reported in Table 1.

**Table 1:** Demographics of students who completed the survey exploring student perceptions of asking and answering questions (n = 417).

**Gender identity**	
Woman	68.3% (285)
Man	30.7% (128)
Other	0.2% (1)
Declined to state	0.7% (3)
**Race/ethnicity**	
Black or African American	6.7% (28)
Hispanic, Latinx, or Spanish origin	20.4% (85)
White/Caucasian	46.5% (194)
Asian	15.3% (64)
American Indian or Alaskan Native	1.2% (5)
Pacific Islander	0.7% (3)
Other/multiple	6.7% (28)
Declined to state	2.4% (10)
**College generation status**	
First-generation	39.1% (163)
Continuing generation	57.8% (241)
Declined to state	3.1% (13)
**Year in college**	
First year	23.7% (99)
Second year	36.5% (152)
Third year	22.3% (93)
Fourth year or beyond	17.5% (73)
Declined to state	0.0% (0)
**Frequency of fear of negative evaluation**	
All the time	12.2% (51)
Most of the time	21.1% (88)
Sometimes	36.7% (153)
Rarely	23.0% (96)
Never	7.0% (29)
Declined to state	0.0% (0)
**Comfort speaking English**	
Extremely comfortable	88.2% (368)
Moderately comfortable	7.4% (31)
Slightly comfortable	3.1% (13)
Slightly uncomfortable	1.0% (4)
Moderately uncomfortable	0.0% (0)
Extremely uncomfortable	0.2% (1)
Declined to state	0.0% (0)
**Average GPA**	3.50

### 1. To what extent do undergraduates perceive that others asking questions in front of large-enrollment college science courses is helpful?

**Fig 1:**
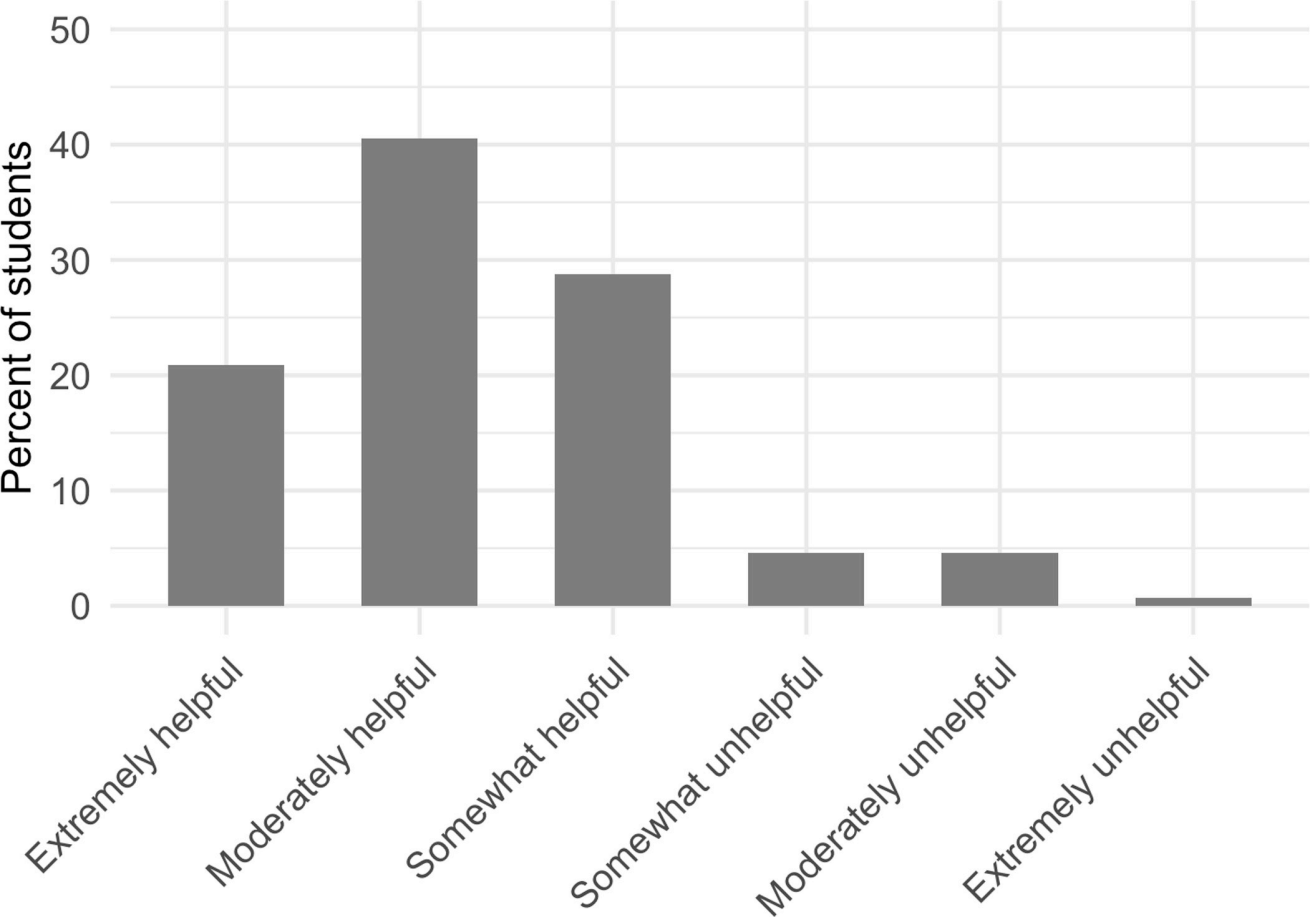
Extent to which students perceive that other students asking instructors questions in front of large-enrollment college science classes is helpful.

Over 90% of students reported that they felt other students voluntarily asking the instructor questions in front of their large-enrollment college science courses was helpful, while only 9.8% of students perceived it to be unhelpful (Fig 1). We tested whether there were demographic differences between students who perceived that other students asking questions was helpful or unhelpful and found that first-generation college students were 6.7x more likely to perceive that other students asking questions was helpful compared to continuing generation college students. We did not identify any other significant demographic differences based on the factors that we tested. The results of this regression to detect demographic differences are reported in the S1 Appendix.

#### Why do students perceive that hearing other students ask questions in front of large-enrollment college science courses is helpful?

Of the students who reported that they perceive other students asking questions in large-enrollment college science courses to be helpful, students most commonly reported that they feel it is helpful because they sometimes have the same question as the one that other students ask in front of the whole class (96.0%). For every one-point increase in fear of negative evaluation, students were 2.5x more likely to select this reason. The next most common response was that students perceived asking questions to be helpful because other students’ questions could help clarify their thinking (83.2%). Seventy-seven percent of students reported that they sometimes felt uncomfortable asking questions themselves; for every one-point increase in FNE, students were 2.0x more likely to report this reason and women were 2.8x more likely to report this reason than men. Students also agreed that they perceived others asking questions as helpful because it gave them a different way of thinking about the material (73.1%). For every one-point increase in GPA, students were 2.0x more likely to select this reason. Finally, 39.1% of students reported that others asking questions was helpful because it broke up the monotony of the lecture. A summary of all of these results is presented in Table 2. The results from the logistic regressions testing to what extent student demographics predict whether a student would select a particular reason for why they perceived it could be helpful for other students to ask questions in large-enrollment courses are presented in the S1 Appendix.

**Table 2:** Percent of students and demographic differences in which students selected each reason why they perceive other students asking questions in front of large-enrollment college science classes is helpful to them.

Reasons students perceive other students asking questions is helpful to them	% (n) N = 376	Summary of significant demographic differences in which students selected each reason
I sometimes have the same question.	96.0% (361)	Students with high fear of negative evaluation (FNE) were more likely to select this reason.
Other students’ questions sometimes help me clarify my thinking.	83.2% (313)	-
I sometimes feel uncomfortable asking questions myself.	77.7% (292)	Women and students with high FNE were more likely to select this reason.
Other students’ questions sometimes give me a different way of thinking about the material.	73.1% (275)	Students with higher GPAs were more likely to select this reason.
Other students’ questions sometimes break up the lecture, which keeps me engaged in class.	39.1% (147)	-

Logistic regression was used to test to what extent student demographics predict whether a student would select a particular reason for why they perceived other students asking questions in large-enrollment college science courses is helpful. The results of all regressions are reported in the S1 Appendix. All significant findings are summarized in the last column of the table. If a box on the table is left blank, no significant demographic differences were found based on the demographic variables we tested. We only included students in these analyses who reported that they felt other students asking questions is helpful (n = 376). No student reported that none of the presented reasons applied to them.

### 2. How frequently do undergraduates report asking questions in front of large-enrollment college science courses?

**Fig 2:**
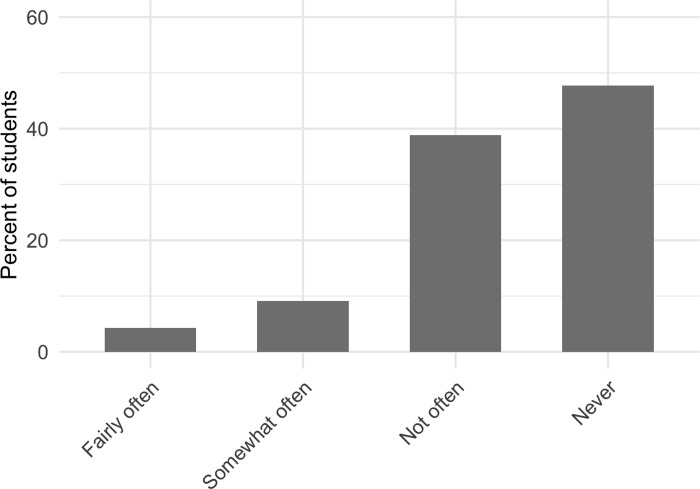
Student self-report of the average number of questions they ask in a large-enrollment college science course per semester.

Nearly half of the students who we surveyed reported that, on average, they never ask questions in their large-enrollment college science courses during the semester (47.7%). In total, only 52.3% of students reported asking at least one question throughout the whole semester; 38.8% reported asking questions not often (1–2 questions per semester), 9.1% reported asking questions somewhat often (3–4 questions per semester), and only 4.3% of students reported asking questions often (5 or more questions per semester) (Fig 2). Women were 2.4x more likely than men to report, on average, never asking a question during large-enrollment college science courses throughout a semester. We did not identify any other significant demographic differences. The results of the regression to detect demographic differences are reported in the S1 Appendix.

#### What discourages students from asking questions in large-enrollment college science courses?

Despite the majority of students perceiving that asking questions is helpful to them, nearly half of students admitted to never volunteering to ask a question and an additional 38.8% of students reported asking only 1–2 questions, on average, per semester in college science courses. As such, we were interested in what discouraged students from asking questions. The most frequently selected factor discouraging students from asking questions to the instructor in front of large-enrollment college science courses is that students feel anxious when they ask questions (72.3%). Women were 3.9x more likely than men to select this reason. Additionally, for every one-point increase in FNE, students were 2.8x more likely to select this reason. Sixty-two percent of students reported that they are discouraged from asking questions because they worry others will judge them; for every one-point increase in FNE, students were 2.2x more likely to select this reason. Over half of students selected that they are discouraged from asking questions because they have the option of asking other students their question during class (57.3%), because they can look up the answer to the question themselves (56.8%), or because they do not know the material well enough (53.1%). Women were 1.8x more likely than men to select that they are discouraged from asking questions because they do not know the material well enough. Additionally, for every one-point increase in FNE, students were 1.6x more likely to select that they did not know the material well enough to ask a question. Nearly 51% of students reported being discouraged from asking questions in front of the whole class because they have the option of asking the instructor the question outside of class. For every one-point increase in GPA, a student was 2.0x more likely to select this reason. Thirty-two percent of students refrained from asking questions because they perceived it would take away from other students’ class time. Twenty-three percent of students reported that another student would likely ask their question and for every one-point increase in FNE, students were 1.5x more likely to select this reason. Twenty-one percent of students reported that they did not think they would get a detailed enough answer to their question during class due to limited time. Latinx students were 2.7x more likely than white students to select that they did not think they would get a detailed enough answer to their question. A summary of these results is presented in Table 3 and results from the logistic regressions testing to what extent student demographics predict whether a student would select a particular factor that discouraged them from asking questions are reported in the S1 Appendix.

**Table 3:** Of students who report on average never asking questions or not often asking questions, the percent and demographic differences in who selected each factor that discourages them from asking questions in large-enrollment college science courses.

Factors that discourage students from asking questions in large-enrollment college science courses	% (n) (N = 361)	Summary of significant demographic differences in which students selected each factor
I feel anxious when I ask questions.	72.3% (261)	Women and students with high fear of negative evaluation (FNE) were more likely to select this reason.
I worry others will judge me.	62.0% (224)	Students with high FNE were more likely to select this reason.
I have the option of asking my questions to other students during class.	57.3% (207)	-
I can look up the answer to my question myself.	56.8% (205)	-
I don’t know the material well enough to ask a good question.	53.1% (192)	Women, and students with high FNE were more likely to select this reason.
I have the option of asking the instructor my question outside of class.	50.7% (183)	Students with higher GPAs were more likely to select this reason.
It would take away from other students’ class time.	32.4% (117)	-
Another student will likely ask my question.	23.6% (85)	Students with high FNE were more likely to select this reason.
I don’t think I will get a detailed enough answer to my question during class due to limited time.	21.7% (78)	Latinx students were more likely to select this reason compared to white students.

Logistic regression was used to test to what extent student demographics predict whether a student would select a particular factor for why they are discouraged from asking questions in large-enrollment college science courses. The results of all regressions are reported in the S1 Appendix. All significant findings are summarized in the last column of the table. If a box on the table is left blank, no significant demographic differences were found based on the demographic variables that we tested. We only included students in these analyses who reported, on average, never asking questions in large-enrollment college science courses or asking only 1–2 questions, on average (n = 361). Four students reported that none of the factors applied to them.

### 3. To what extent do undergraduates perceive that others answering questions in front of large-enrollment college science courses is helpful?

**Fig 3:**
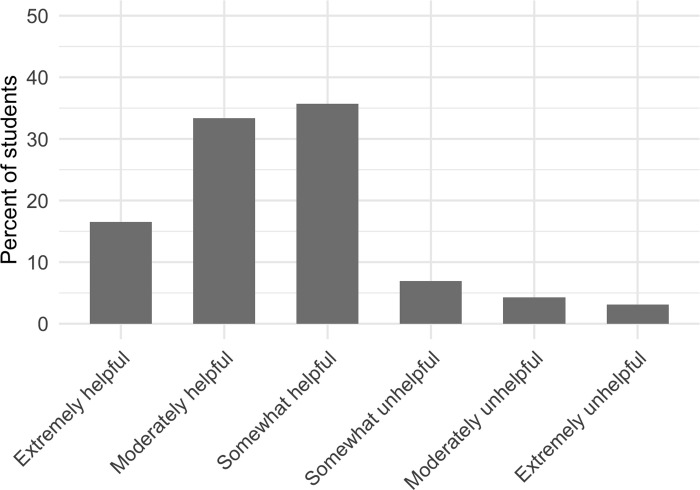
The extent to which students report they perceive other students answering instructor questions in large-enrollment college science courses is helpful.

Over 86% of students reported that they perceived it to be helpful when other students voluntarily answer instructor questions in front of the large-enrollment college science courses (Fig 3). Specifically, 16.5% of students reported that they found other students answering questions to be extremely helpful, 33.3% found it moderately helpful, and 35.7% found it somewhat helpful. Conversely, 3.1% of students found other students answering questions to be extremely unhelpful, 4.3% found it to be moderately unhelpful, and 7.0% reported it to be somewhat unhelpful. There were no demographic differences between students who perceived answering questions as helpful and those who perceived it as unhelpful. The results of the regression to detect demographic differences are reported in the S1 Appendix.

#### Why do students perceive other students answering instructor questions in front of large-enrollment college science courses is helpful?

Of the students who perceived other students voluntarily answering questions in front of large-enrollment college science courses to be helpful, students most commonly reported that they perceive others answering questions to be helpful because they can sometimes gain knowledge from other students’ responses (89.1%) and because other students sometimes have different ways of explaining information (77.9%). For every additional year a student had spent in college, they were 1.5x more likely to report that other students have different ways of explaining information. Nearly 75% of students reported that they perceived others answering questions to be helpful because instructors’ responses can sometimes provide helpful details. Additionally, a majority of students (58.5%) selected that they perceive that other students answering questions is helpful because other students’ answers can allow them to check their own understanding of the material. Further, 42.6% of students said that they perceive that other students answering instructor questions is helpful because if other students are wrong, then it can show them that it is okay for them to be wrong too. Asian students were 2.0x more likely than white students to report this reason. Additionally, for every one-point increase in FNE, a student was 1.5x more likely to select that they perceive it is helpful when other students answer questions because if they are wrong, then it can show them that it is okay for them to be wrong too. Finally, 26.1% of students reported that they found it helpful when others answer questions because it breaks up the lecture and keeps them more engaged in class. A summary of results is presented in Table 4. All results from the logistic regressions testing to what extent student demographics predict whether a student would select a particular reason for why they perceive other students answering instructor questions in large-enrollment college science courses is helpful are reported in the S1 Appendix.

**Table 4:** Percent of students and demographic differences in who selected each reason they perceive other students answering instructor questions in front of large-enrollment college science classes is helpful to them.

Reasons students perceive other students answering questions is helpful to them	% (n) (N = 357)	Summary of significant demographic differences in which students selected each reason
I can sometimes gain knowledge from other students’ responses.	89.1% (318)	-
Other students sometimes have different ways of explaining information.	77.9% (278)	Students who had spent more time in college were more likely to select this reason.
An instructor’s response can sometimes provide helpful details.	74.8% (267)	-
Other students’ answers sometimes allow me to check my own understanding of the material.	58.5% (209)	-
If another student is wrong, it sometimes shows me that it is okay for me to be wrong too.	42.6% (152)	Asian students and students who have high fear of negative evaluation (FNE) were more likely to select this reason.
Other students answering questions sometimes breaks up the lecture, which keeps me engaged in class	26.1% (93)	-

Logistic regression was used to test to what extent student demographics predict whether a student would select a particular reason for why they perceived other students answering questions in large-enrollment college science courses is helpful. The results of all regressions are reported in the S1 Appendix. All significant findings are summarized in the last column of the table. If a box on the table is left blank, no significant demographic differences were found based on the demographic variables that we tested. We only included students in these analyses who reported that they felt other students answering questions is helpful (n = 357). Five students reported that none of the presented reasons applied to them.

### 4. How frequently do undergraduates report answering questions in front of large-enrollment college science courses?

**Fig 4:**
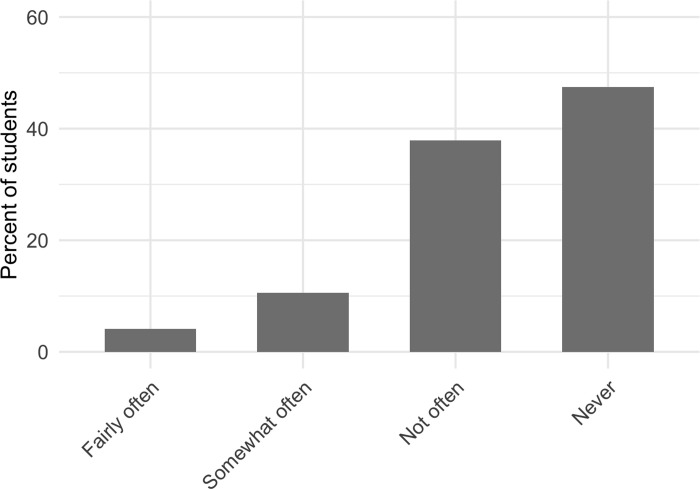
The extent to which students report the average number of questions they answer in a large-enrollment college science course per semester.

Nearly half of students (47.5%) reported that, on average, they never voluntarily answer questions in large-enrollment college science courses when there are opportunities to do so. Conversely, 52.5% of students reported that, on average, they answer at least one question per semester in large-enrollment college science courses; 37.9% reported that they do not answer questions often (1–2 questions per semester), 10.6% of students said that they answer questions somewhat often (3–4 questions per semester), and 4.1% of students reported answering questions fairly often (5 or more questions per semester) (Fig 4). For every one-point increase in FNE, students were 1.3x more likely to report that, on average, they never voluntarily answer questions in large-enrollment college science courses.

#### What discourages students from answering questions in front of large-enrollment college science courses?

Of the students who reported never answering questions or not often answering questions, the most frequently selected factor discouraging students from answering instructor questions is that students are not confident that their answers are correct (75.6%). Women were 2.8x more likely than men to select this reason and for every one-point increase in FNE, students were 1.4x more likely to select this factor. Students also commonly selected that they feel anxious when answering instructors’ questions (66.0%); for every one-point increase in FNE, students were 2.3x more likely to select this reason. Over half of students (57.6%) reported that they worry other students will judge them. Compared to Asian students, white students were 2.9x more likely to select this factor and first-generation college students were 1.9x more likely to report this compared to continuing generation college students. Additionally, for every one-point increase in FNE, students were 2.8x more likely to report this factor. Twenty-seven percent of students selected that they were discouraged from answering instructor questions because someone else will likely answer the question, so they do not have to be the one to share the answer. Finally, 20.5% of students reported that they were discouraged from answering questions because it would take away from other students’ class time. A summary of results is presented in Table 5 and results from the logistic regressions testing to what extent student demographics predict whether a student would select a particular factor that discourages them from answering questions are reported in the S1 Appendix.

**Table 5:** Of students who report on average never answering questions or not often answering questions, the percent of and demographic differences in who selected each factor that discourages them from answering questions in large-enrollment college science courses.

Factors that discourage students from answering questions in large-enrollment college science courses	% (n) (N = 356)	Summary of significant demographic differences in which students selected each factor
I am not confident that my answer is correct.	75.6% (269)	Women and students with high FNE were more likely to select this reason.
I feel anxious when I answer instructors’ questions.	66.0% (235)	Students with high FNE were more likely to select this reason.
I worry other students will judge me.	57.6% (205)	White students (compared to Asian students), first-generation college students and students with high FNE were more likely to select this reason.
Someone else will likely answer the question, so I don’t have to.	27.2% (97)	-
It would take away from other students’ class time.	20.5% (73)	-

Logistic regression was used to test to what extent student demographics predict whether a student would select a particular factor for why they are discouraged from answering questions in large-enrollment college science courses. The results of all regressions are reported in the S1 Appendix. All significant findings are summarized in the last column of the table. If a box on the table is left blank, no significant demographic differences were found based on the demographic variables we tested. We only included students in these analyses who reported, on average, never answering or answering 1–2 questions per semester in large-enrollment college science courses (n = 356). Twenty students reported that none of the factors applied to them.

## Discussion

**Fig 5:**
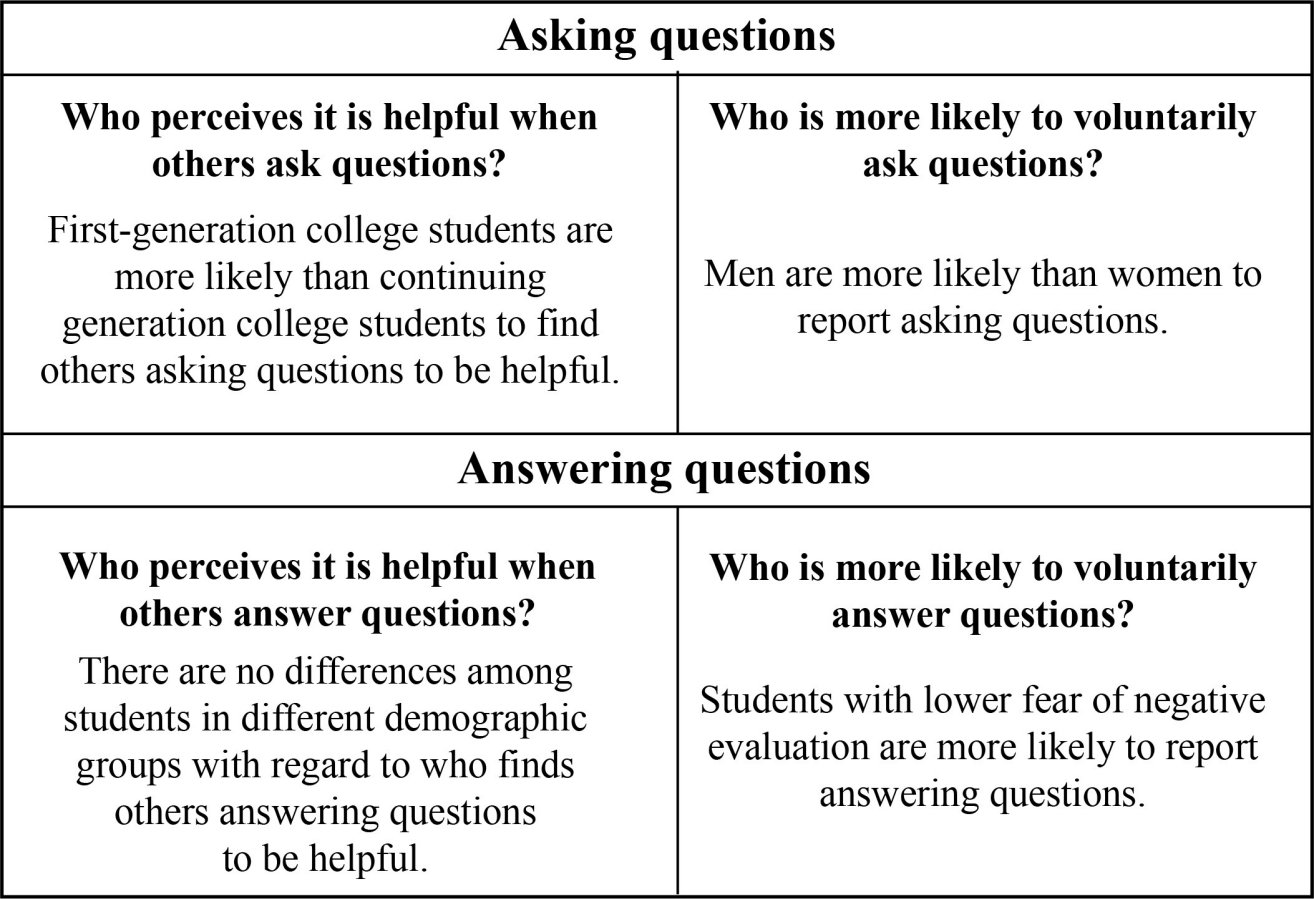
A summary of demographic differences with regard to which students found asking and answering questions most helpful and which students reported answering and asking questions most often.

In this study we examined student perceptions of two specific aspects of participation in large-enrollment college science courses: voluntarily asking questions to an instructor and voluntarily answering instructor-posed questions in front of the whole class. A summary of which students were more likely to find asking and answering questions helpful and which students reported answering and asking questions most frequently are reported in Fig 5. Despite literature suggesting that students may be confused by other students’ questions and answers [26] or exasperated by peers who contribute too much to whole-class discussion [5], we found that students overwhelmingly reported that other students asking and answering questions was helpful to them. Notably, compared to continuing generation college students, first-generation college students were more likely to report that they found other students asking questions in large-enrollment college science courses to be helpful. Studies taking a student-deficit approach suggest that first-generation students come into the college science classroom knowing less about college than their peers whose parents or guardians attended college [61]. As such, these studies would support the notion that first-generation students may find questions, particularly questions about course expectations or questions about how to succeed in the course, especially helpful [62]. However, other work suggests that first-generation students have unique assets; specifically, one study found that first-generation students use strategic thinking when needing to find answers, suggesting they are metacognitively aware [63]. As such, first-generation students may be more metacognitive in class, and thus benefit more from listening to other students ask questions than their continuing generation peers. When we examined whether there were demographic differences in why students perceived other students asking questions to be helpful, there were no discernable differences between first-generation college students and continuing generation college students. However, future studies examining students’ perceptions of the types of questions (e.g. questions about science, questions about course expectations) may shed light as to why first-generation college students perceive other students asking questions to be more helpful than their continuing generation peers.

Despite students’ overwhelming agreement that other students asking and answering questions was helpful to them, only about half of students reported voluntarily asking questions and voluntarily answering questions in their large-enrollment college science courses. We found that men were more likely than women to report voluntarily asking questions in large-enrollment college science courses. With regard to answering questions, we did not find any gender differences. These results are partly consistent with findings from a recent observational study of 34 courses showing that men are more likely to participate in life sciences courses [34]. However, this study did not examine whether there were gender differences based on the unique practices of either asking or answering questions. Another recent observational study that was able to disaggregate these practices found that men were disproportionately likely to both ask and answer questions in large-enrollment biology courses [10]. Two other observational studies reported findings opposite of those of our study; they found men and women were equally likely to ask questions, but that men were more likely than women to voluntarily answer questions [11,12]. All of these studies highlight a gender gap in course participation, but vary with regard to what practices result in participation differences. These inconsistencies in results may be due to factors such as instructor gender, immediacy of the instructor, what percentage of the class was comprised of women, or general classroom climate; alternatively, these contrasting results could be due to differences in methodology [21,34,64–67]. While observational studies can provide more exact insight into student behavior, we chose to take a self-report approach to this study because it negated the need to interpret student identities from physical appearance alone (e.g. students were able to identify their gender instead of us as observers having to guess their gender). As such, this approach allowed us to explore patterns with identities and characteristics that tend to be less visible than gender, such as college generation status and student year in school.

Another factor that influenced student-reported participation in our study was fear of negative evaluation (FNE), which is the primary emotion underlying student anxiety in courses that engage students in whole-class participation, such as asking and answering questions in front of the whole class [28,55,68]. Rather than correlating generalized anxiety with student behavior, identifying what specific underlying emotion drives student differences is an important step in examining how to increase student participation and make courses more equitable. Historically, FNE has been primarily studied in language-learning courses where students are regularly asked to participate in class and studies have shown that students’ FNE discourages them from speaking in class and can cause them to make mistakes when they do speak in class [69,70]. Recently, discipline-based education researchers have begun to examine the role of FNE in college science courses. While qualitative studies have shown that students who fear that others may judge their responses are reluctant to participate in both large- and small-enrollment college science courses [28,68], studies have found that FNE is particularly high for students in large-enrollment courses [71,72]. However, this relationship has never been explored quantitatively in the sciences. As such, we incorporated FNE into our models assessing students’ perceptions of asking and answering questions. Not only did students’ levels of FNE negatively predict whether they reported answering a question in class in our study, it also predicted the factors that discouraged students from both asking and answering questions. Students with higher FNE reported that they were discouraged from both asking and answering questions because they were concerned about being judged for their response and because the process of either asking or answering a question made them feel anxious. Both students’ perception of judgement and their anxiety have been shown to be highly related to FNE [28,68,73]. Additionally, students with higher FNE reported that they did not know the material well enough to ask a good question and that they were not confident enough in their response to answer a question. Given that our model controlled for GPA as a proxy for student preparedness, it is unlikely that these students with higher FNE knew the material any less well than students with lower FNE. However, this finding corroborates other studies that have shown that students who unfavorably evaluate themselves are more likely to expect to be negatively evaluated by others [28,74]. Therefore, bolstering students’ self-efficacy or their perception of their ability to do well in science courses, particularly compared to others, is likely important in encouraging student participation [10,41,42].

### Limitations

This study was conducted at a single R1 institution and focused on large-enrollment college science classes. As such, these findings may not be generalizable to other institution types (e.g. master’s granting, primarily undergraduate institutions, community colleges), class types (e.g. small-enrollment courses), or types of courses (e.g. humanities, business). Additionally, while we recruited students across science courses, biology majors were overrepresented in our sample, which could have biased our results. However, we know of no literature which suggests that biology majors feel or behave differently than their peers in other science majors with regard to asking and answering questions. Additionally, the students who chose to participate in this survey collectively had a higher-than-average GPA (3.5) than the collective average GPA of science students at the institution (~3.3). As such, these findings may be more representative of higher achieving students. Finally, we asked students to report, on average, how often they voluntarily asked and answered questions in college science courses. It is possible that students’ self-recollection of their behavior was inaccurate and may not reflect the behaviors students actually exhibited during their classes. While an observational study would have provided more precise insight to students’ behavior, we chose to have students self-report their behaviors because it allowed us to explore whether other non-visible identities (e.g. college generation status) influenced students’ participation.

## Conclusion

In this study, we examined to what extent students found it helpful when other students voluntarily ask and answer questions in large-enrollment college science courses and why. We also explored to what extent students reported asking and answering questions in large-enrollment college science courses and what factors discouraged them from doing so. We found that, overwhelmingly, students reported that other students asking and answering questions is helpful to them and that first-generation students were more likely than continuing generation students to report other students asking questions is helpful. We also found that nearly half of students report that they do not ask questions during a semester, with women being more likely to report never asking questions compared to men. Additionally, nearly half of students report never answering questions during the semester. In sum, while students perceive voluntarily asking and answering questions to be a helpful classroom practice, nearly half choose to not participate in these practices in large-enrollment college science courses. The primary reason that students choose not to ask questions seems to be related to their anxiety, while the primary reason that students do not answer questions seems to be their lack of confidence; fear of negative evaluation is an underlying construct that could explain both of these findings. This work provides insight into important factors that affect student engagement in large-enrollment college science courses.

## Supporting information

S1 Appendix Supplementary material. All supplementary material for the manuscript including a copy of the survey and results of additional analyses.
(DOCX)

S1 Dataset Manuscript dataset. Dataset used in all data analyses.
(XLSX)
