# Supplementary material for: Call on me! Undergraduates’ perceptions of voluntarily asking and answering questions in front of large-enrollment science classes
Source: PLoS One. 2021 Jan 12;16(1):e0243731. doi: 10.1371/journal.pone.0243731 (PMC7802933; doi:10.1371/journal.pone.0243731)
Supplement: S1 Appendix — All supplementary material for the manuscript including a copy of the survey and results of additional analyses. (DOCX) [file pone.0243731.s001.docx]

S1 Appendix 1 for

***Call on me! Undergraduates’ perceptions of voluntarily asking and answering questions in front of large-enrollment science classes***

Corresponding author: Katelyn M. Cooper

This supplement contains the following:

Item Page

Preliminary interview questions 3

Demographics of interview participants 5

Number of students who participated in survey 5

Copy of survey questions analyzed 6

Results of open-coding for why students perceive asking questions to be helpful 11

Results of open-coding for why students perceive answering questions to be helpful 13

Results of open-coding for what discourages students from asking questions 15

Results of open-coding for what discourages students from answering questions 18

Results of logistic regression testing whether there are demographic differences between students who perceive asking questions as helpful vs unhelpful (1 table) 20

Results of logistic regressions testing whether there are demographic differences between students who select particular factors as to why asking questions is helpful (5 tables) 21

Results of logistic regression testing whether there are demographic differences between students who do and do not ask questions (1 table) 23

Results of logistic regressions testing whether there are demographic differences between students who select particular factors that discourage them from asking questions (9 tables) 24

Results of logistic regression testing whether there are demographic differences between students who perceive answering questions as helpful vs unhelpful (1 table) 28

Results of logistic regressions testing whether there are demographic differences between students who select particular factors as to why answering questions is helpful (6 tables) 29

Results of logistic regression testing whether there are demographic differences between students who do and do not answer questions (1 table) 32

Results of logistic regressions testing whether there are demographic differences between students who select particular factors that discourage them from answering questions (5 tables)

33

**Preliminary interview questions**

***About asking questions***

Were you ever enrolled in large-enrollment college science courses where students were able to voluntarily ask questions during class?

What is your opinion of when students voluntarily ask questions during your large-enrollment science courses?

How, if at all, has hearing other students voluntarily ask questions to the instructor during your large-enrollment science courses advantaged or helped you personally?

How, if at all, has hearing other students voluntarily ask questions to the instructor during your large-enrollment science courses disadvantaged you personally?

To what extent do you personally volunteer to ask instructor questions during your large-enrollment science courses?

Why do you ask questions or why do you not ask questions?

If you ask questions:

Can you think of any (additional) advantages of you personally asking questions during your large-enrollment science courses? Please explain your reasoning.

Can you think of any (additional) disadvantages of you personally asking questions during your large-enrollment science courses? Please explain your reasoning.

***About answering questions***

Were you ever enrolled in large-enrollment college science courses where students were able to answer questions to the instructor in front of the whole course?

What is your opinion of when students voluntarily answer instructor questions during your large-enrollment science courses?

How, if at all, has hearing other students voluntarily ask questions to the instructor during your large-enrollment science courses advantaged or helped you personally?

How, if at all, has hearing other students voluntarily answer instructor questions during your large-enrollment science courses disadvantaged you personally?

To what extent do you personally volunteer to answer instructor questions during your large-enrollment science courses?

Why do you answer questions or why do not answer questions?

Can you think of any (additional) advantages (if you were to) of you personally volunteering to answer instructor questions during your large-enrollment science courses? Please explain your reasoning.

Can you think of any (additional) disadvantages (if you were to) of you personally volunteering to answer instructor questions during your large-enrollment science courses? Please explain your reasoning.

**Demographics of interview participants**

**Table 1.** Demographics of students who completed the pilot interviews (n=50).

| **Gender identity** | |
| --- | --- |
| Female | 66% (33) |
| Male | 32% (16) |
| Other | 2% (1) |
| Declined to state | 0% (0) |
| **Race/ethnicity** | |
| Black or African American | 2% (1) |
| Hispanic, Latinx, or Spanish origin | 14% (7) |
| White/Caucasian | 50% (25) |
| Asian | 18% (9) |
| American Indian or Alaskan Native | 0% (0) |
| Pacific Islander | 0% (0) |
| Other/multiple selected | 14% (7) |
| Declined to state | 2% (1) |
| **Average GPA (4 pt. scale)** | 3.61 |
| **Student major** | |
| Biology | 88% (44) |
| Double Major | 10% (5) |
| Other | 2% (1) |
| Declined to state | 0% (0) |

**Number of students who participated in survey**

**Table 2.** Number of students who participated and completed the survey and which science class they were recruited from at the R1 institution (n=417).

| **Biology** | |
| --- | --- |
| BIO I | 94 |
| BIO II | 189 |
| **Total** | 283 |
| **Chemistry** | |
| CHM I | 33 |
| CHM II | 62 |
| CHM III | 7 |
| **Total** | 102 |
| **Physics** | |
| PHY I | 11 |
| PHY II | 3 |
| **Total** | 14 |
| **Did not report class** | 18 |

**Copy of survey questions analyzed**

1) About how many large-enrollment science courses have you been enrolled in during your time in college?

o 1-2

o 3-4

o 5-6

o 7-8

o 9-10

o 11 or more

o I have never been enrolled in a large-enrollment science course.

2) Have you ever been, or are you currently, enrolled in a large-enrollment science course where students can voluntarily ASK the instructor questions in front of the whole class?

o Yes

o No

**Hearing other students voluntarily ASK the instructor a question**

3) To what extent do you feel it is helpful when you hear other students voluntarily ask the instructors of large-enrollment science courses questions in front of the whole class?

o Extremely helpful

o Moderately helpful

o Slightly helpful

o Slightly unhelpful

o Moderately unhelpful

o Extremely unhelpful

4) Please provide reasons why hearing other students voluntarily ask instructors of large-enrollment science courses questions in front of the whole class could be helpful to you?

5) Please provide reasons why hearing other students voluntarily ask instructors of large-enrollment science courses questions in front of the whole class could be unhelpful to you?

6) Please choose all the reasons why hearing other students voluntarily ask instructors of large-enrollment science courses questions in front of the whole class could be helpful to you. Please select all that apply.

▢ I sometimes have the same question, so it is helpful to hear the answer.

▢ I sometimes feel uncomfortable asking questions myself, so it is helpful when other students ask the question I have so I don't have to.

▢ Other students' questions sometimes give me a different way of thinking about the material.

▢ Other students' questions sometimes help me clarify my thinking.

▢ Other students' questions sometimes break up the lecture, which keeps me engaged in class.

▢ None of these apply to me.

7) Please choose all the reasons why hearing other students voluntarily ask instructors of large-enrollment science courses questions in front of the whole class could be unhelpful to you. Please select all that apply.

▢ Other students' questions sometimes take up class time.

▢ Other students' questions sometimes disrupt the flow of class.

▢ Other students' questions sometimes make me feel like I'm less smart.

▢ Other students' questions sometimes cause me to be confused.

▢ Other students' questions can sometimes cause the class to run late.

▢ If someone else asks a question, sometimes there is not enough time for my own question.

▢ None of these apply to me.

**Personally ASKING the instructor a question**

8) On average, how often throughout a semester do you voluntarily ask questions to instructors of large-enrollment science courses in front of the whole class?

o Never (0 questions per semester)

o Not often (1-2 questions per semester)

o Somewhat often (3-4 questions per semester)

o Fairly often (5 or more questions per semester)

9) What factors could discourage you from voluntarily asking questions to instructors of large-enrollment science courses in front of the whole class?

10) Please choose all of the factors that could discourage you from voluntarily asking questions to instructors of large-enrollment science courses in front of the whole class. Please select all that apply.

▢ I worry others will judge me.

▢ I feel anxious when I ask questions.

▢ It would take away from other students' class time.

▢ I don't know the material well enough to ask a good question.

▢ Another student will likely ask my question.

▢ I have the option of asking the instructor my question outside of class.

▢ I have the option of asking my questions to other students during class.

▢ I can look up the answer to my question myself.

▢ I don't think I will get a detailed enough answer to my question during class due to limited time.

▢ None of these apply to me.

**Hearing other students voluntarily ANSWER questions**

11) Have you ever been, or are you currently, enrolled in a large-enrollment science course where students can voluntarily ANSWER instructor questions in front of the whole class?

o Yes

o No

12) To what extent do you think that it is helpful when you hear other students voluntarily answer questions from instructors of large-enrollment science courses in front of the whole class?

o Extremely helpful

o Moderately helpful

o Slightly helpful

o Slightly unhelpful

o Moderately unhelpful

o Extremely unhelpful

13) Please provide reasons why hearing other students voluntarily answer questions from instructors of large-enrollment science courses in front of the whole class could be helpful to you?

14) Please provide reasons why hearing other students voluntarily answer questions from instructors of large-enrollment science courses in front of the whole class could be unhelpful to you?

15) Please choose all the reasons why hearing other students voluntarily answer questions from instructors of large-enrollment science courses in front of the whole class could be helpful to you. Please select all that apply.

▢ I can sometimes gain knowledge from other students' responses.

▢ Other students sometimes have different ways of explaining information.

▢ An instructor's response can sometimes provide helpful details.

▢ Other students' answers sometimes allow me to check my own understanding of the material.

▢ If another student is wrong, it sometimes shows me that it is okay for me to be wrong too.

▢ Other students answering questions sometimes breaks up the lecture, which keeps me engaged in class.

▢ None of these apply to me.

16) Please choose all the reasons why hearing other students voluntarily answer questions from instructors of large-enrollment science courses in front of the whole class could be unhelpful to you. Please select all that apply.

▢ Other students' answers sometimes make me feel less smart.

▢ Other students' answers sometimes take up class time.

▢ Other students' answers sometimes distract me.

▢ Other students' answers sometimes make me more confused.

▢ Sometimes other students are rude when answering questions.

▢ None of these apply to me.

**Personally ANSWERING instructor questions**

17) On average, how often throughout a semester do you voluntarily answer questions from instructors of large-enrollment science courses in front of the whole class?

o Never (0 questions per semester)

o Not often (1-2 questions per semester)

o Somewhat often (3-4 questions per semester)

o Fairly often (5 or more questions per semester)

18) What factors could discourage you from voluntarily answering questions from instructors of large-enrollment science courses in front of the whole class?

19) Please choose all of the factors that could discourage you from voluntarily answering questions from instructors of large-enrollment science courses in front of the whole class. Please select all that apply.

▢ I worry other students will judge me.

▢ I feel anxious when I answer instructors' questions.

▢ It would take away from other students' class time.

▢ I am not confident that my answer is correct.

▢ Someone else will likely answer the question, so I don't have to.

▢ None of these apply to me.

**Demographic questions**

20) I most closely identify as

o Woman

o Man

o Non-binary

o Decline to state

21) I most closely identify as

o American Indian or Alaska Native

o Asian

o Black or African American

o Hispanic, Latinx, or Spanish origin

o Pacific Islander

o White/Caucasian

o Other (please describe)

o Decline to state

22) What is your parent or guardian's highest level of education? If you have more than one parent or guardian with differing levels of education, choose the higher of the two.

o Less than high school completed

o High school diploma or GED

o Some college but no degree

o Associate's degree (for example: AA, AS)

o Bachelor's degree (for example: BA, AB, BS)

o Master's degree (for example: MA, MS, MEng, MEd, MSW, MBA)

o Higher than a Master's degree (for example: PhD, MD, JD)

o Decline to state

23) How comfortable are you speaking English in front of others?

o Extremely comfortable

o Moderately comfortable

o Slightly comfortable

o Slightly uncomfortable

o Moderately uncomfortable

o Extremely uncomfortable

24) What is your GPA (on a 4.0 scale)?

25) How long have you attended college while pursuing your undergraduate degree?

o 1 year or less

o 2 years

o 3 years

o 4 years

o 5 years or more

o I have graduated with my undergraduate degree

26) To what extent do you worry that others in your college science courses will judge you when you interact with them?

o All the time

o Most of the time

o Sometimes

o Rarely

o Never

**Results of open-coding of the open-ended question students were asked about why they perceive other students voluntarily asking questions in large-enrollment college science courses to be helpful.**

Five researchers (E.A., B.B., W.D.B.T., M.R.C., and I.F.) used open-coding methods to identify common themes among students’ responses to the open-ended question about why students perceive other students voluntarily asking questions in large-enrollment college science courses to be helpful. Each individual member of this research team reviewed 100 responses from students using inductive coding and took detailed analytic notes (Birks & Mills, 2015). The researchers then convened to compare codes and develop one preliminary coding rubric. Each researcher then coded a set of 40 responses using the group rubric; they came together and compared their codes and revised their rubric. The researchers used constant comparison methods to verify that quotes within a category were similar to one another and not too different to warrant the creation of a new theme (Glesne & Peshkin, 1992). This process was repeated until the group was confident with their rubric. Once a final rubric was established, all researchers individually used the rubric to code the entire data set. They compared their codes for each student and agreed upon a final set of codes. Each theme is mutually exclusive, that is an excerpt of text could only be coded as one theme. However, students’ full responses often included multiple themes.

**Table 3.** Reasons why students perceive other students asking instructor questions in large-enrollment college science courses to be helpful, the percent of students who reported each theme, and one example quote.

| **Theme** | **Percent**  **(n/365)** | **Description** | **Student example quote** |
| --- | --- | --- | --- |
| **Similar question** | **55.9%**  (204/365) | Students describe that they think other students asking questions is helpful because they had a similar or the same question in mind. | "They can be helpful to me because if I am not understanding something, but I don't know how to ask the question, the other person could ask it in a better way and get me the answer I need." |
| **Elaboration/clarification** | **35.6%**  (130/365) | Students describe that they think other students asking questions is helpful because it provides more clarity on subjects which will clear up any confusions the students may have. In addition, students mention it is helpful because it will reiterate and reinforce concepts. | "It is great to hear other students ask instructors questions because it can help me know if I am unclear about a specific detail in a topic. Being able to hear a concept again from either me asking a question or a peer asking a question helps to root in the details." |
| **Discomfort** | **19.7%**  (72/365) | Students describe that they think other students asking questions is helpful because they want to ask a question themselves but they feel too shy, afraid or nervous to ask. | "Normally I'm too anxious to ask a question myself so it is nice if someone else asks a question because 9/10 times I won't." |
| **Novel question** | **16.2%**  (59/365) | Students describe that they think other students asking questions is helpful because they had not thought about that question or concept before. | "Everyone thinks differently so if someone asks a question, I may have never thought of that so it is beneficial to me to hear their question being answered." |
| **Novel perspective** | **9.3%**  (34/365) | Students describe that they think other students asking questions is helpful because it provides them with a new perspective or a new way of thinking about the material. | "Other people will have different ways of looking at the same problem, which can give me more insight into the problem and help me solve it, thus bettering my understanding of the underlying concepts." |
| **Comfort** | **4.7%**  (17/365) | Students describe that they think other students asking questions is helpful because it will make them feel more comfortable to do so themselves or that they no longer feel alone in their confusion. | "Seeing them ask questions makes it easier for me to ask questions because it makes me more comfortable knowing that I am not the only one who did not understand something." |
| **Un-codable** | **8.5%**  (31/365) | The student did not clearly answer the question, or the student's response was not able to be categorized using the themes above. | NA |

Overall, the categories of students’ open-ended responses closely reflected the factors presented to students in the closed-ended question, with one exception. Eleven students did not respond.

**Results of open-coding of the open-ended question students were asked about why they perceive other students voluntarily answering questions in large-enrollment college science courses to be helpful.**

Five researchers (J.O.G.R., G.G., S.G.P., Y. I. R. C., and K.S.) used open-coding methods to identify common themes among students’ responses to the open-ended question about why students perceive other students voluntarily answering questions in large-enrollment college science courses to be helpful. Each individual member of this research team reviewed 100 responses from students using inductive coding and took detailed analytic notes (Birks & Mills, 2015). The researchers then convened to compare codes and develop one preliminary coding rubric. Each researcher then coded a set of 40 responses using the group rubric; they came together and compared their codes and revised their rubric. The researchers used constant comparison methods to verify that quotes within a category were similar to one another and not too different to warrant the creation of a new theme (Glesne & Peshkin, 1992). This process was repeated until the group was confident with their rubric. Once a final rubric was established, all researchers individually used the rubric to code the entire data set. They compared their codes for each student and agreed upon a final set of codes. Each theme is mutually exclusive, that is an excerpt of text could only be coded as one theme. However, students’ full responses often included multiple themes.

**Table 4.** Reasons why students perceive other students answering instructor questions in large-enrollment college science courses to be helpful, the percent of students who reported each theme, and one example quote.

| **Theme** | **Percent (n/343)** | **Description** | **Student example quote** |
| --- | --- | --- | --- |
| **Novel perspective/ clarification** | **56.3%** (193/343) | Students describe that they think other students answering questions is helpful because they think it helps them clarify, learn, and solidify course content. The answer provides easier to comprehend explanations, simple wording, and it may provide a different perspective. | “It can provide answers that are creative or unorthodox, and from a perspective that might understand how students learn better than a teacher.” |
| **Class comparison** | **17.5%** (60/343) | Students describe that they think other students answering questions is helpful because they think using the answers provided by their peers to compare their own knowledge and evaluate if they need to study more for the class is helpful. | “It provides a good check for where I am at and possibly a different perspective that allows me to challenge my current knowledge.” |
| **Classroom variety** | **8.5%** (29/343) | Students describe that they think other students answering questions is helpful because it breaks up the lecture, makes students feel more engaged and interested with the course material, makes class time feel shorter, and allows students to think critically about a problem. Also allows for a break to think about response. | “It adds variety to the class instead of hearing only the professor speak through the whole thing.” |
| **Instructor feedback/instructor clarification** | **7.0%** (24/343) | Students describe that they think other students answering questions is helpful because the instructor expands on the material, corrects any misconceptions, and expands on topics the class is struggling with. | “They may not get the entire topic as a whole so the instructor can help guide them to the right way of thinking.” |
| **Class representative** | **5.2%** (18/343) | Students describe that they think other students answering questions is helpful especially when a peer speaks on behalf of other students when students feel nervous or uncomfortable to answer themselves. Also, they may provide the response multiple students had in their minds. | “Other students may provide the answers I was going to but did not, so it’s almost as if I answered the question!” |
| **Un-codable** | **18.7%** (64/343) | The student did not clearly answer the question, or the student's response was not able to be categorized using the themes above. | NA |

The categories of students’ open-ended responses closely reflected the factors presented to students in the closed-ended question. Fourteen students did not respond.

**Results of open-coding of the open-ended question students were asked about what discourages them from voluntarily asking questions in large-enrollment college science courses.**

Five researchers (S.A.F., A.E.H., A.H., M.L.K., and N.J.W.) used open-coding methods to identify common themes among students’ responses to the open-ended question about what discourages them from voluntarily asking questions in large-enrollment college science courses. Each individual member of this research team reviewed 100 responses from students using inductive coding and took detailed analytic notes (Birks & Mills, 2015). The researchers then convened to compare codes and develop one preliminary coding rubric. Each researcher then coded a set of 40 responses using the group rubric; they came together and compared their codes and revised their rubric. The researchers used constant comparison methods to verify that quotes within a category were similar to one another and not too different to warrant the creation of a new theme (Glesne & Peshkin, 1992). This process was repeated until the group was confident with their rubric. Once a final rubric was established, all researchers individually used the rubric to code the entire data set. They compared their codes for each student and agreed upon a final set of codes. Each theme is mutually exclusive, that is an excerpt of text could only be coded as one theme. However, students’ full responses often included multiple themes.

**Table 5.** Reasons why students are discouraged from voluntarily asking questions in large-enrollment college science courses, the percent of students who reported each theme, and one example quote.

| **Theme** | **Percent (n/196)** | **Description** | **Student example quote** |
| --- | --- | --- | --- |
| **Fear of negative evaluation from other students (FNE)** | **29.6%** (58/196) | Students describe being discouraged from asking because of feeling dumb/stupid asking a question. Students worry others will judge them, that they will be perceived of as less smart, dumb, stupid, or that they will sound ignorant if they ask a bad question.  Any time a student describes worrying about others evaluating or judging them, it is FNE. | “What discourages me from asking questions is that I think other students are judging me, or if my question is stupid to ask.” |
| **Discomfort with public speaking** | **21.9%** (43/196) | Students describe being discouraged from asking not because of feeling anxious but rather because of feeling timid or shy. Students describe not liking projecting their voice (this mostly has to do with volume). They do not want to be the center of attention. Students state they lack self-confidence and are naturally uncomfortable speaking in class. | “I can be a shy person so I'm not very good at public speaking.” |
| **Anxiety/insecurity** | **19.9%** (39/196) | Students describe being discouraged from asking because of feeling nervous about speaking because of a large class size. Students also report having social anxiety and are intimidated by speaking. Additionally, they can describe that they are nervous that their question may have already been answered (but if they are worried others will judge them, this is FNE). They also fear a follow up question will be asked. The student can describe that they are nervous or scared. | “I feel intimidated in a room of 100 plus students more so when there is a microphone involved so I prefer to let other brave students answer.” |
| **Wasting other’s time** | **19.4%** (38/196) | Students describe being discouraged from asking because their question will waste the time of the professor and other students. They are worried about disrupting others and believe their question would break up the lecture. They are also worried their question will cause the instructor to fall behind and rush the rest of the lecture.  If a student worries that their questions are not important enough *because* it would disrupt others, that is coded here, not in anxiety. | “The main factor is just slowing the class down. If I ask a question the teacher will have to explain the answer and it will slow the other 100 or 200 kids down.” |
| **Student preference** | **15.3%** (30/196) | Students describe being discouraged from asking because they would rather find out the information on their own, go to office hours, email the professor, or ask after class. Students prefer one-on-one instruction; they may ask their peers or individuals outside of their normal class time. They may also prefer to find the answer themselves after class. | “I feel that I can figure out the answer by reviewing class material or looking it up online. I sometimes wait to ask a professor until the end of class because I feel that my question is individual, not something everyone needs to hear.” |
| **Class size** | **11.2%** (22/196) | Students describe being discouraged from asking because they do not like large class sizes without stating any specific emotion(s) associated with volume (nervousness, being uncomfortable, etc.). | “There’s just too many people. These courses should be kept to 75 people at most, in my opinion.” |
| **Unprepared to ask** | **7.7%** (15/196) | Students describe being discouraged from asking because they feel that they are completely lost and too confused about the material to inquire about anything during class time. They also worry that they are too unprepared to ask a question regarding the class material. They state that their lack of attention during class restricts their understanding and therefore makes them unable to ask questions associated with the lecture material. | “Most of the time I don't pay attention in class so I never have anything I would be confused about.” |
| **Professor response/reaction** | **7.7%** (15/196) | Students describe being discouraged from asking because they are worried their professor will respond negatively, will be rude or will not fully answer the question. Professor is dismissive of student’s questions. They also worry that the professor will not see them raising their hand to ask a question. | “When professors are rude about answering the  question or make you feel like you shouldn't ask the question in the first place.” |
| **Un-codable** | **3.6%** (7/196) | The student did not clearly answer the question, or the student's response was not able to be categorized using the themes above. | NA |

Overall, the categories of students’ open-ended responses closely reflected the factors presented to students in the closed-ended question, with a few exceptions. Students’ open-ended responses highlighted that they were discouraged from asking questions because they were shy or did not want to be the center of attention, because of the large class size, or because the professor may respond negatively. Only the responses of students who reported never asking questions were analyzed; three of those students did not respond to this open-ended question.

**Results of open-coding of the open-ended question students were asked about what discourages them from voluntarily answering questions in large-enrollment college science courses.**

Four researchers (F.S., C.F.S., K.S., and K.W.) used open-coding methods to identify common themes among students’ responses to the open-ended question about what discourages them from voluntarily answering questions in large-enrollment college science courses. Each individual member of this research team reviewed 100 responses from students using inductive coding and took detailed analytic notes (Birks & Mills, 2015). The researchers then convened to compare codes and develop one preliminary coding rubric. Each researcher then coded a set of 40 responses using the group rubric; they came together and compared their codes and revised their rubric. The researchers used constant comparison methods to verify that quotes within a category were similar to one another and not too different to warrant the creation of a new theme (Glesne & Peshkin, 1992). This process was repeated until the group was confident with their rubric. Once a final rubric was established, all researchers individually used the rubric to code the entire data set. They compared their codes for each student and agreed upon a final set of codes. Each theme is mutually exclusive, that is an excerpt of text could only be coded as one theme. However, students’ full responses often included multiple themes.

**Table 6.** Reasons why students are discouraged from voluntarily answering questions in large-enrollment college science courses, the percent of students who reported each theme, and one example quote.

| **Theme** | **Percent (n/191)** | **Description** | **Student example quote** |
| --- | --- | --- | --- |
| **Fear of negative evaluation from other students (FNE)** | **40.8%** (78/191) | Students describe being discouraged from answering because they fear being wrong or judged by their peers. | “The fear that it might be an easy question being asked and I do not want to get it wrong. The feeling that other students may judge you for your answer or response.” |
| **Uncomfortable answering** | **24.1%** (46/191) | Students describe being discouraged from answering because they are usually uncomfortable participating in front of others, which can involve projecting their voice and being more visible in the classroom. | “I feel uncomfortable when pressure is put on me to answer a particular question out loud in front of others...” |
| **Unprepared to answer** | **16.8%** (32/191) | Students describe being discouraged from answering when they feel unprepared to answer, especially relating to new information being covered in class. | “I am not familiar with the material before class which discourages me from answering the questions.” |
| **Does not see value in answering** | **7.3%** (14/191) | Students describe being discouraged from answering because they have a negative attitude towards classroom participation, some believe students answering questions is a waste of time and prefer learning in other ways. | “I feel student answers are generally less helpful than the instructor simply giving me information. I do not feel the need to prove my intelligence to others.” |
| **Class size** | **5.8%** (11/191) | Students describe being discouraged from answering when they are intimidated by the size of their large-enrollment science courses. | “The large class size makes me feel very self-conscious.” |
| **Fear of negative evaluation from instructor (FNE)** | **4.7%**  (9/191) | Students describe being discouraged from answering because they fear being judged by their instructor who may appear to be rude or non-welcoming. | “I don't want to say a wrong answer and the teacher make an embarrassing remark about it or laugh.” |
| **Un-codable** | **17.3%** (33/191) | The student did not clearly answer the question, or the student's response was not able to be categorized using the themes above. | NA |

Overall, the categories of students’ open-ended responses closely reflected the factors presented to students in the closed-ended question, with a few exceptions. Students’ open-ended responses highlighted that they were discouraged from answering questions because they worried that they may be judged by others, and mainly because they felt uncomfortable. Only the responses of students who reported never answering questions were analyzed; seven of those students did not respond to this open-ended question.

**Results of logistic regression testing whether there are demographic differences between students who perceive asking questions as helpful vs unhelpful (1 table)**

helpful.ask.binary ~ gender + race + gen.stat + year + FNE + gpa

Coefficients:

Estimate Std. Error z value Pr(>|z|)

(Intercept) 4.66401 2.15999 2.16 0.03083 *

genderwoman 0.00231 0.43588 0.01 0.99577

raceasian 0.39933 0.53956 0.74 0.45924

racelatinx -0.72007 0.45707 -1.58 0.11516

gen.statfgen 1.90239 0.53675 3.54 0.00039 ***

year -0.23053 0.20231 -1.14 0.25450

FNE -0.16266 0.17963 -0.91 0.36520

gpa -0.54299 0.49098 -1.11 0.26875

**Results of logistic regressions testing whether there are demographic differences between students who select particular factors as to why asking questions is helpful (5 tables)**

**I sometimes have the same question**

help.ask.same ~ gender + race + gen.stat + year + FNE + gpa

Coefficients:

Estimate Std. Error z value Pr(>|z|)

(Intercept) -0.8292 3.1850 -0.26 0.795

genderwoman 0.9352 0.6053 1.55 0.122

raceasian 0.8132 0.8501 0.96 0.339

racelatinx 0.0315 0.7911 0.04 0.968

gen.statfgen 0.3380 0.6699 0.50 0.614

year 0.0201 0.2992 0.07 0.946

FNE 0.9123 0.3248 2.81 0.005 **

gpa 0.1567 0.7712 0.20 0.839

**Other students’ questions help me clarify my thinking**

help.ask.clarify ~ gender + race + gen.stat + year + FNE + gpa

Coefficients:

Estimate Std. Error z value Pr(>|z|)

(Intercept) 1.5620 1.7027 0.92 0.359

genderwoman -0.1502 0.3703 -0.41 0.685

raceasian -0.4211 0.4084 -1.03 0.302

racelatinx 0.2241 0.4325 0.52 0.604

gen.statfgen -0.4369 0.3476 -1.26 0.209

year -0.2017 0.1646 -1.23 0.220

FNE 0.2774 0.1577 1.76 0.079 .

gpa 0.0232 0.4028 0.06 0.954

**I sometimes feel uncomfortable asking questions myself**

help.ask.uncomfortable ~ gender + race + gen.stat + year + FNE + gpa

Coefficients:

Estimate Std. Error z value Pr(>|z|)

(Intercept) -1.4175 1.7189 -0.82 0.4096

genderwoman 1.0424 0.3291 3.17 0.0015 **

raceasian -0.3939 0.3820 -1.03 0.3025

racelatinx 0.1924 0.4521 0.43 0.6704

gen.statfgen 0.0824 0.3490 0.24 0.8134

year 0.1665 0.1687 0.99 0.3236

FNE 0.6927 0.1666 4.16 3.2e-05 ***

gpa -0.0637 0.4091 -0.16 0.8762

**Other students’ questions give me a different way of thinking about the material**

help.ask.material ~ gender + race + gen.stat + year + FNE + gpa

Coefficients:

Estimate Std. Error z value Pr(>|z|)

(Intercept) -1.1654 1.3895 -0.84 0.402

genderwoman -0.2296 0.3105 -0.74 0.460

raceasian -0.5184 0.3442 -1.51 0.132

racelatinx 0.5698 0.3560 1.60 0.109

gen.statfgen -0.3401 0.2833 -1.20 0.230

year 0.0711 0.1366 0.52 0.603

FNE -0.0423 0.1290 -0.33 0.743

gpa 0.6801 0.3296 2.06 0.039 *

**Other students’ questions sometimes break up lecture and keep me engaged**

help.ask.engaged ~ gender + race + gen.stat + year + FNE + gpa

Coefficients:

Estimate Std. Error z value Pr(>|z|)

(Intercept) 0.0581 1.2949 0.04 0.96

genderwoman 0.4276 0.2840 1.51 0.13

raceasian -0.1115 0.3325 -0.34 0.74

racelatinx 0.2756 0.3075 0.90 0.37

gen.statfgen 0.1055 0.2610 0.40 0.69

year 0.1458 0.1255 1.16 0.25

FNE -0.1742 0.1189 -1.47 0.14

gpa -0.1776 0.3044 -0.58 0.56

**Results of logistic regression testing whether there are demographic differences between students who do and do not ask questions (1 table)**

frequency.ask.never ~ gender + race + gen.stat + year + FNE + gpa

Coefficients:

Estimate Std. Error z value Pr(>|z|)

(Intercept) 0.64059 1.22520 0.523 0.601080

genderwoman -0.89009 0.26976 -3.300 0.000968 ***

raceasian 0.09333 0.31405 0.297 0.766323

racelatinx -0.19481 0.28930 -0.673 0.500695

gen.statfgen 0.38971 0.25071 1.554 0.120082

year -0.09913 0.11932 -0.831 0.406105

FNE -0.14073 0.10907 -1.290 0.196976

gpa 0.15318 0.28760 0.533 0.594297

**Results of logistic regressions testing whether there are demographic differences between students who select particular factors that discourage them from asking questions (9 tables)**

**I feel anxious when I ask questions**

discourage.ask.anxious ~ gender + race + gen.stat + year + FNE + gpa

Coefficients:

Estimate Std. Error z value Pr(>|z|)

(Intercept) -1.7670 1.7016 -1.04 0.30

genderwoman 1.3685 0.3398 4.03 5.7e-05 ***

raceasian -0.0646 0.4125 -0.16 0.88

racelatinx -0.4616 0.4008 -1.15 0.25

gen.statfgen 0.0108 0.3436 0.03 0.97

year 0.1056 0.1704 0.62 0.54

FNE 1.0344 0.1719 6.02 1.8e-09 ***

gpa -0.3581 0.3984 -0.90 0.37

**I worry others will judge me**

discourage.ask.judge ~ gender + race + gen.stat + year + FNE + gpa

Coefficients:

Estimate Std. Error z value Pr(>|z|)

(Intercept) -2.0542 1.4483 -1.42 0.16

genderwoman 0.0925 0.3184 0.29 0.77

raceasian -0.5380 0.3580 -1.50 0.13

racelatinx 0.5902 0.3675 1.61 0.11

gen.statfgen 0.0144 0.2977 0.05 0.96

year -0.1599 0.1458 -1.10 0.27

FNE 0.8081 0.1457 5.55 2.9e-08 ***

gpa 0.1553 0.3359 0.46 0.64

**I can look up the answer to the question myself**

discourage.ask.look ~ gender + race + gen.stat + year + FNE + gpa

Coefficients:

Estimate Std. Error z value Pr(>|z|)

(Intercept) 0.1191 1.2660 0.09 0.93

genderwoman -0.0571 0.2878 -0.20 0.84

raceasian -0.2754 0.3328 -0.83 0.41

racelatinx -0.1904 0.3002 -0.63 0.53

gen.statfgen -0.4004 0.2613 -1.53 0.13

year -0.1368 0.1263 -1.08 0.28

FNE 0.1185 0.1142 1.04 0.30

gpa 0.1143 0.2949 0.39 0.70

**I have the option of asking my questions to other students in class**

discourage.ask.during ~ gender + race + gen.stat + year + FNE + gpa

Coefficients:

Estimate Std. Error z value Pr(>|z|)

(Intercept) -0.8133 1.2746 -0.64 0.52

genderwoman -0.1789 0.2918 -0.61 0.54

raceasian -0.2753 0.3336 -0.83 0.41

racelatinx 0.3226 0.3078 1.05 0.29

gen.statfgen -0.4199 0.2632 -1.60 0.11

year 0.0497 0.1273 0.39 0.70

FNE 0.0743 0.1150 0.65 0.52

gpa 0.3156 0.2972 1.06 0.29

**I don’t know the material well enough to ask a good question**

discourage.ask.material ~ gender + race + gen.stat + year + FNE + gpa

Coefficients:

Estimate Std. Error z value Pr(>|z|)

(Intercept) 0.5772 1.3385 0.43 0.66633

genderwoman 0.5986 0.2988 2.00 0.04513 *

raceasian -0.4414 0.3476 -1.27 0.20420

racelatinx 0.3475 0.3179 1.09 0.27445

gen.statfgen -0.5135 0.2770 -1.85 0.06374 .

year -0.0401 0.1331 -0.30 0.76343

FNE 0.4693 0.1232 3.81 0.00014 ***

gpa -0.5934 0.3150 -1.88 0.05956 .

**I have the option of asking the instructor a question outside of class**

discourage.ask.outside ~ gender + race + gen.stat + year + FNE + gpa

Coefficients:

Estimate Std. Error z value Pr(>|z|)

(Intercept) -2.1825 1.2892 -1.69 0.090 .

genderwoman 0.4004 0.2914 1.37 0.169

raceasian 0.2072 0.3386 0.61 0.541

racelatinx 0.2180 0.3058 0.71 0.476

gen.statfgen -0.4000 0.2624 -1.52 0.127

year 0.0928 0.1278 0.73 0.468

FNE -0.1914 0.1156 -1.66 0.098 .

gpa 0.6758 0.3021 2.24 0.025 *

**It would take away from other students’ class time**

discourage.ask.away ~ gender + race + gen.stat + year + FNE + gpa

Coefficients:

Estimate Std. Error z value Pr(>|z|)

(Intercept) -1.2472 1.3558 -0.92 0.36

genderwoman -0.2294 0.2958 -0.78 0.44

raceasian 0.2637 0.3424 0.77 0.44

racelatinx 0.3623 0.3203 1.13 0.26

gen.statfgen -0.4412 0.2797 -1.58 0.11

year 0.0656 0.1347 0.49 0.63

FNE -0.1647 0.1205 -1.37 0.17

gpa 0.3312 0.3167 1.05 0.30

**Another student will likely ask my question**

discourage.ask.likely ~ gender + race + gen.stat + year + FNE + gpa

Coefficients:

Estimate Std. Error z value Pr(>|z|)

(Intercept) -2.1361 1.5132 -1.41 0.1580

genderwoman -0.1849 0.3364 -0.55 0.5825

raceasian 0.0397 0.3886 0.10 0.9187

racelatinx -0.0825 0.3619 -0.23 0.8197

gen.statfgen -0.2844 0.3118 -0.91 0.3618

year -0.0664 0.1505 -0.44 0.6593

FNE 0.3814 0.1382 2.76 0.0058 **

gpa 0.0370 0.3491 0.11 0.9157

**I don’t think I will get a detailed enough answer to my question during class due to limited time**

discourage.ask.limit ~ gender + race + gen.stat + year + FNE + gpa

Coefficients:

Estimate Std. Error z value Pr(>|z|)

(Intercept) -2.7782 1.5793 -1.76 0.079 .

genderwoman 0.1293 0.3611 0.36 0.720

raceasian 0.3721 0.4158 0.89 0.371

racelatinx 0.9781 0.3562 2.75 0.006 **

gen.statfgen -0.5401 0.3343 -1.62 0.106

year 0.2295 0.1564 1.47 0.142

FNE 0.1924 0.1402 1.37 0.170

gpa 0.0169 0.3597 0.05 0.962

**Results of logistic regression testing whether there are demographic differences between students who perceive answering questions as helpful vs unhelpful (1 table)**

helpful.answer.binary ~ gender + race + gen.stat + year + FNE + gpa

Coefficients:

Estimate Std. Error z value Pr(>|z|)

(Intercept) 4.5015 1.7832 2.52 0.012 *

genderwoman -0.2341 0.3746 -0.62 0.532

raceasian 0.1566 0.4245 0.37 0.712

racelatinx 0.1383 0.4120 0.34 0.737

gen.statfgen 0.3565 0.3516 -1.01 0.311

year -0.0326 0.1665 -0.20 0.845

FNE -0.0108 0.1503 -0.07 0.943

gpa -0.6624 0.4255 -1.56 0.120

**Results of logistic regressions testing whether there are demographic differences between students who select particular factors as to why answering questions is helpful (6 tables)**

**I can sometimes gain knowledge from other students’ responses**

help.answer.knowledge ~ gender + race + gen.stat + year + FNE + gpa

Coefficients:

Estimate Std. Error z value Pr(>|z|)

(Intercept) 1.6160 2.1445 0.75 0.45

genderwoman -0.0197 0.4741 -0.04 0.97

raceasian -0.1015 0.5717 -0.18 0.86

racelatinx -0.3138 0.5080 -0.62 0.54

gen.statfgen -0.0681 0.4516 -0.15 0.88

year -0.1031 0.2105 -0.49 0.62

FNE 0.2558 0.2024 1.26 0.21

gpa 0.0794 0.5149 0.15 0.88

**Other students sometimes have different ways of explaining information**

help.answer.different ~ gender + race + gen.stat + year + FNE + gpa

Coefficients:

Estimate Std. Error z value Pr(>|z|)

(Intercept) -0.5020 1.5481 -0.32 0.75

genderwoman 0.3864 0.3294 1.17 0.24

raceasian -0.5668 0.3746 -1.51 0.13

racelatinx 0.1923 0.4031 0.48 0.63

gen.statfgen -0.2398 0.3280 0.73 0.46

year 0.3759 0.1611 2.33 0.02 *

FNE 0.2020 0.1477 1.37 0.17

gpa 0.0063 0.3859 0.02 0.99

**An instructor’s response can sometimes provide helpful details**

help.answer.details ~ gender + race + gen.stat + year + FNE + gpa

Coefficients:

Estimate Std. Error z value Pr(>|z|)

(Intercept) -0.860891 1.408763 -0.61 0.541

genderwoman -0.269721 0.326809 -0.83 0.409

raceasian 0.297770 0.387635 0.77 0.442

racelatinx 0.356794 0.356315 1.00 0.317

gen.statfgen 0.068051 0.300958 -0.23 0.821

year -0.000911 0.141163 -0.01 0.995

FNE 0.227008 0.136710 1.66 0.097 .

gpa 0.370371 0.345055 1.07 0.283

**Other students’ answers allow me to check my understanding**

help.answer.understanding ~ gender + race + gen.stat + year + FNE + gpa

Coefficients:

Estimate Std. Error z value Pr(>|z|)

(Intercept) -0.9322 1.2706 -0.73 0.46

genderwoman 0.1414 0.2831 0.50 0.62

raceasian 0.0393 0.3363 0.12 0.91

racelatinx 0.1923 0.3142 0.61 0.54

gen.statfgen -0.0347 0.2686 0.13 0.90

year 0.1089 0.1269 0.86 0.39

FNE 0.0941 0.1204 0.78 0.43

gpa 0.1779 0.3101 0.57 0.57

**If another student is wrong, it sometimes shows me that it is okay for me to be wrong too**

help.answer.wrong ~ gender + race + gen.stat + year + FNE + gpa

Coefficients:

Estimate Std. Error z value Pr(>|z|)

(Intercept) -1.3840 1.3021 -1.06 0.2878

genderwoman 0.5299 0.2983 1.78 0.0757 .

raceasian 0.6929 0.3466 2.00 0.0456 *

racelatinx 0.4950 0.3159 1.57 0.1171

gen.statfgen 0.4045 0.2723 -1.49 0.1373

year 0.0689 0.1301 0.53 0.5966

FNE 0.3835 0.1259 3.05 0.0023 **

gpa -0.1696 0.3160 -0.54 0.5915

**Other students answering questions sometimes breaks up the lecture, which keeps me engaged in class**

help.answer.engaged ~ gender + race + gen.stat + year + FNE + gpa

Coefficients:

Estimate Std. Error z value Pr(>|z|)

(Intercept) -1.5142 1.3901 -1.09 0.28

genderwoman 0.3701 0.3217 1.15 0.25

raceasian 0.1798 0.3696 0.49 0.63

racelatinx 0.0730 0.3365 0.22 0.83

gen.statfgen 0.1746 0.2917 -0.60 0.55

year 0.0990 0.1374 0.72 0.47

FNE -0.0276 0.1311 -0.21 0.83

gpa 0.0563 0.3379 0.17 0.87

**Results of logistic regression testing whether there are demographic differences between students who do and do not answer questions (1 table)**

frequency.answer.never ~ gender + race + gen.stat + year + FNE + gpa

Coefficients:

Estimate Std. Error z value Pr(>|z|)

(Intercept) 1.2876 1.2056 1.07 0.286

genderwoman -0.3795 0.2632 -1.44 0.149

raceasian -0.0838 0.3080 -0.27 0.786

racelatinx -0.1654 0.2851 -0.58 0.562

gen.statfgen 0.1145 0.2459 0.47 0.642

year -0.0917 0.1175 -0.78 0.435

FNE -0.2556 0.1084 -2.36 0.018 *

gpa 0.0118 0.2824 0.04 0.967

**Results of logistic regressions testing whether there are demographic differences between students who select particular factors that discourage them from answering questions (5 tables)**

**I am not confident that my answer is correct**

discourage.answer.confident ~ gender + race + gen.stat + year + FNE + gpa

Coefficients:

Estimate Std. Error z value Pr(>|z|)

(Intercept) -1.62125 1.52094 -1.066 0.28644

genderwoman 1.03209 0.32423 3.183 0.00146 **

raceasian -0.72132 0.38138 -1.891 0.05858 .

racelatinx 0.06200 0.39303 0.158 0.87465

gen.statfgen -0.28454 0.32603 -0.873 0.38279

year -0.01928 0.15448 -0.125 0.90068

FNE 0.31136 0.14404 2.162 0.03065 *

gpa 0.43650 0.36015 1.212 0.22552

**I feel anxious when I answer instructors’ questions**

discourage.answer.anxious ~ gender + race + gen.stat + year + FNE + gpa

Coefficients:

Estimate Std. Error z value Pr(>|z|)

(Intercept) -3.8470 1.5090 -2.549 0.0108 *

genderwoman 0.4836 0.3221 1.501 0.1332

raceasian -0.3703 0.3740 -0.990 0.3222

racelatinx 0.3842 0.3790 1.014 0.3108

gen.statfgen 0.3262 0.3098 1.053 0.2924

year 0.1054 0.1502 0.701 0.4831

FNE 0.8436 0.1530 5.514 3.51e-08 ***

gpa 0.3821 0.3458 1.105 0.2692

**I worry other students will judge me**

discourage.answer.judge ~ gender + race + gen.stat + year + FNE + gpa

Coefficients:

Estimate Std. Error z value Pr(>|z|)

(Intercept) -4.3973447 1.5094402 -2.913 0.00358 **

genderwoman -0.0484536 0.3331874 -0.145 0.88438

raceasian -1.0527537 0.3886152 -2.709 0.00675 **

racelatinx 0.0009083 0.3685499 0.002 0.99803

gen.statfgen 0.6314622 0.3081676 2.049 0.04045 *

year -0.0251017 0.1499197 -0.167 0.86703

FNE 1.0426957 0.1614534 6.458 1.06e-10 ***

gpa 0.4931614 0.3441229 1.433 0.15183

**Someone else will likely answer the question so I don’t have to**

discourage.answer.likely ~ gender + race + gen.stat + year + FNE + gpa

Coefficients:

Estimate Std. Error z value Pr(>|z|)

(Intercept) -1.89112 1.27326 -1.485 0.137

genderwoman -0.03954 0.29132 -0.136 0.892

raceasian 0.01592 0.33887 0.047 0.963

racelatinx -0.27266 0.30616 -0.891 0.373

gen.statfgen -0.15990 0.26166 -0.611 0.541

year 0.11257 0.12599 0.893 0.372

FNE 0.06826 0.11541 0.591 0.554

gpa 0.40069 0.29871 1.341 0.180

**It would take away from other students’ class time**

discourage.answer.time ~ gender + race + gen.stat + year + FNE + gpa

Coefficients:

Estimate Std. Error z value Pr(>|z|)

(Intercept) -0.72040 1.49016 -0.483 0.6288

genderwoman -0.61122 0.33044 -1.850 0.0644 .

raceasian 0.19539 0.40173 0.486 0.6267

racelatinx 0.69346 0.35589 1.949 0.0514 .

gen.statfgen -0.34095 0.32127 -1.061 0.2886

year 0.01267 0.15116 0.084 0.9332

FNE 0.02496 0.13674 0.183 0.8551

gpa -0.08279 0.34964 -0.237 0.8128
